# Real-Time Monitoring of Cisplatin-Induced Cell Death

**DOI:** 10.1371/journal.pone.0019714

**Published:** 2011-05-16

**Authors:** Hamed Alborzinia, Suzan Can, Pavlo Holenya, Catharina Scholl, Elke Lederer, Igor Kitanovic, Stefan Wölfl

**Affiliations:** Institute of Pharmacy and Molecular Biotechnology, Heidelberg University, Heidelberg, Germany; Massachusetts Institute of Technology, United States of America

## Abstract

Since the discovery of cisplatin more than 40 years ago and its clinical introduction in the 1970s an enormous amount of research has gone into elucidating the mechanism of action of cisplatin on tumor cells. With a novel cell biosensor chip system allowing continuous monitoring of respiration, glycolysis, and impedance we followed cisplatin treatment of different cancer cell lines in real-time. Our measurements reveal a first effect on respiration, in all cisplatin treated cell lines, followed with a significant delay by interference with glycolysis in HT-29, HCT-116, HepG2, and MCF-7 cells but not in the cisplatin-resistant cell line MDA-MB-231. Most strikingly, cell death started in all cisplatin-sensitive cell lines within 8 to 11 h of treatment, indicating a clear time frame from exposure, first response to cisplatin lesions, to cell fate decision. The time points of most significant changes were selected for more detailed analysis of cisplatin response in the breast cancer cell line MCF-7. Phosphorylation of selected signal transduction mediators connected with cellular proliferation, as well as changes in gene expression, were analyzed in samples obtained directly from sensor chips at the time points when changes in glycolysis and impedance occurred.

Our online cell biosensor measurements reveal for the first time the time scale of metabolic response until onset of cell death under cisplatin treatment, which is in good agreement with models of p53-mediated cell fate decision.

## Introduction

Cisplatin has been used in cancer chemotherapy for more than 30 years against different human tumors, since its approval by the FDA in 1978 [1], [2]. DNA is the proven primary target of cisplatin, and cisplatin adduct formation effects many DNA-dependent cellular functions, including inhibition of replication and transcription, cell cycle arrest, and DNA damage leading to cell death and apoptosis, but may also result in mutations [3], [4], [5], [6]. Despite clarity about the basic mechanism of cisplatin toxicity leading to induce cell death in sensitive cells [3], it still remains unclear how cisplatin triggers cell death over time in a cell population. We used a cell biosensor chip system for continuous monitoring of changes in cell metabolism and cell morphology for time-resolved analysis of cisplatin action on tumor cells including the breast cancer cell lines MCF-7 (p53 wild type) and MDA-MB-231 (p53 mutant), the colon cancer cell lines HT-29 and HCT-116, and the hepatocellular carcinoma HepG2.

**Figure 1:**
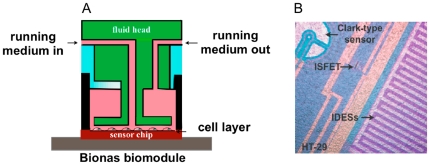
Outline of the cell biosensor chip system. *A*, schematic diagram of the flow system for biosensor chip analysis, showing the inlet and outlet for running medium and the small incubation chamber. *B*, picture of cells (HT29) growing on biosensor chip. Electrode sensors are visible and marked: ISFET: pH sensor; Clark-type sensor: O_2_ sensor; IDESs; interdigitated electrodes for impedance measurements.

The biosensor chip system used (Bionas 2500) allows simultaneous measurement o f several metabolic parameters of the specific cells grown on the biosensor chip (Fig. 1*A*–*B*), in our case (*i*) glycolytic activity measured as pH change, (*ii*) cellular respiration measured as oxygen consumption, and (*iii*) cellular morphology, adhesion, cell–cell interactions, and membrane functionality measured as cellular impedance [7], [8], [9].

Monitoring the cellular response to cisplatin in real time we observed distinct time profiles for changes in respiration, glycolysis, and impedance. The observed drastic change of impedance is a clear indicator for the onset of cell death. Although respiration was the first parameter affected by cisplatin, experiments with isolated mitochondria showed no immediate effect of cisplatin on mitochondrial respiratory activity in a time frame when respiration of intact cells was clearly reduced. At time points of most significant changes in the breast cancer cell line MCF-7 we performed more detailed analysis of signal transduction connected with cell proliferation and of gene expression. Interestingly, no significant change of analyzed pathways could be detected when glycolysis and impedance changes occurred, at 8 and 11 h upon cisplatin treatment, respectively, while after 24 h a decrease in p-Akt1 and p-GSK-3β reflected reduced pro-survival signaling. Pro-apoptotic regulation was visible by changes in gene expression. Expression of several pro-apoptotic genes was already induced when glycolysis changed and much more so when impedance changed at onset of cell death. In contrast, stress response genes were strongly regulated when glycolysis changed but did not show much further induction when impedance changed. This suggests that in response to cisplatin, first an initial general stress response is activated before pro-apoptotic cell fate decision. The time frames observed in our online measurements indicate that this crucial transition occurs at around 10 h upon cisplatin treatment.

## Results

### Changes in cellular metabolism in response to cisplatin

**Figure 2:**
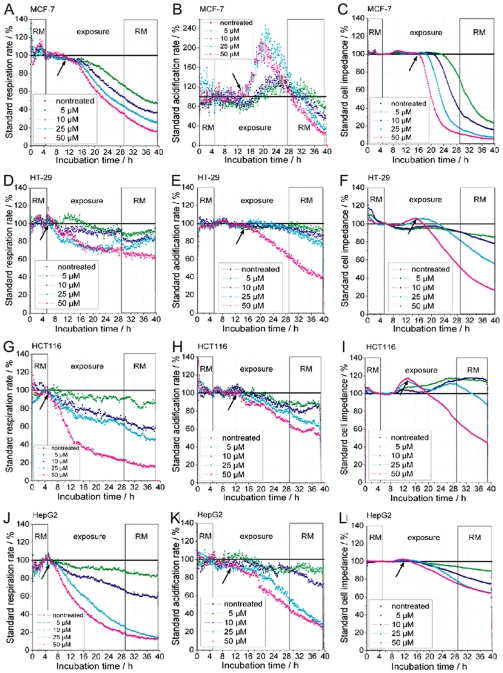
Real-time response profiles of cellular respiration, glycolysis, and impedance upon cisplatin treatment. Cisplatin treatment started after 5 h of equilibration of cancer cell tissue cultures in the biosensor chip system and was continued over 24 h. RM (running medium) marks cisplatin-free medium; exposure marks time when cisplatin was presented at indicated concentrations (cisplatin-free control, 5 µM, 10 µM, 25 µM, 50 µM). *A*,*D*,*G*,*J*, change in respiration (oxygen in the medium), *B*,*E*,*H*,*K*, glycolysis, depicted as change in acidification of the medium (extracellular pH), *C*,*F*,*I*,*L*, change in impedance of the cell layer (potential between interdigitated electrodes) using four cancer cells lines, *A*,*B*,*C*, MCF-7, *D*,*E*,*F*, HT-29, *G*,*H*,*I*, HCT-116, and *J*,*K*,*L*, HepG2. *Arrows* indicate the time points of significant changes at the highest concentration (50 µM) of cisplatin used.

Cisplatin was applied to the cancer cell lines MCF-7, HT-29, HCT-116, and HepG2 and the response measured by the cell Bionas 2500 biosensor system (Fig. 2*A–L*). MCF-7 cells did not respond immediately to cisplatin. It rather took about 5–6 h before cellular respiration decreased, followed by an increase in glycolysis after 8–9 h upon treatment with 50 µM cisplatin. After these metabolic changes a drastic decrease in cellular impedance occurred at about 10–11 h after exposure to cisplatin (Fig. 2 *C*), which reflects the onset of cisplatin induced cell death. High cisplatin concentrations showed a more rapid and severe response. This response profile is very characteristic for MCF-7 cells. The colon carcinoma cells, HT-29 and HCT-116, show a significantly different response. Both cell lines show an immediate decrease in respiration but no significant change in glycolysis before cells started to die, seen by a significant decrease of impedance at about 8–9 h in HCT-116 cells and at about 10–11 h in HT-29 cells (Fig. 2*F*, *I*). Cell death is also reflected in the complete reduction of metabolism, in particular the decrease of glycolysis slightly pre-running the impedance change. The transient increase of impedance visible in HCT-116 cells (Fig. 2*I*) was observed in all experiments and indicates a change in cell interactions in response to the treatment before induction of cell death.

**Figure 3:**
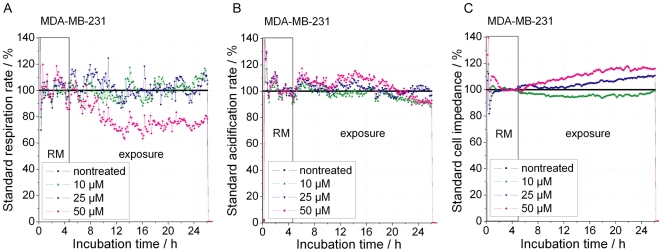
Real-time response profiles of cellular respiration, glycolysis, and impedance upon cisplatin treatment of cisplatin-resistant breast cancer cell line MDA-MB-231. Cisplatin treatment started after 5 h of equilibration of cancer cell tissue cultures in the biosensor chip system and was continued over 20 h. RM (running medium) marks cisplatin free medium; exposure marks the time when cisplatin was presented at the indicated concentrations (cisplatin-free control, 10 µM, 25 µM, 50 µM). *A*, change in respiration (oxygen in the medium). *B*, glycolysis, depicted as change in acidification of the medium (extracellular pH). *C*, change in impedance of the cell layer (potential between interdigitated electrodes) using four cisplatin-resistant breast cancer cell lines MDA-MB-231.

Treatment of HepG2 cells also led to an immediate decrease in respiration and a delayed decrease in glycolysis (Fig. 1*J–L*). Again cells start to die independent of the applied cisplatin concentration after about 8–9 h, although the extent of cell death is concentration dependent. An overview of the response time frame of the above cell lines to 50 µM cisplatin is presented in Table 1. Regarding the cisplatin-resistant breast cancer cell line MDA-MB-231 it should be noted that, while cisplatin had no effect on glycolysis and cellular impedance, a decrease in respiration could be observed at the highest (50 µM) cisplatin concentration, clearly indicating that these cells sense cisplatin but are not sensitive to cisplatin induced cell death (Fig. 3).

**Table 1:** Time points of metabolic and impedance changes in response to 50 µM cisplatin.

Cancer cell line	Respiration change/hour	Glycolysis change/hour	Impedance change/hour
MCF-7	5–6 (Decrease)	*8–9 (**Increase**)10–11 (Decrease)	10–11 (Decrease)
HT-29	Immediate response (Decrease)	9–10 (Decrease)	10–11 (Decrease)
HCT-116	Immediate response (Decrease)	6–7 (Decrease)	*4–5 (**Increase**)8–9 (Decrease)
HepG2	Immediate response (Decrease)	6–7 (Decrease)	8–9 (Decrease)

*Denotes two transitions for one parameter: here, an increase precedes the final decrease of the measured value.

It should be emphasized, however, that none of the sensitive cells lines showed a recovery, even with lower cisplatin concentrations, when cisplatin was removed from the medium after 24 h of treatment (Fig. 2*A–L*).

### Measurement of mitochondrial oxygen consumption

**Figure 4:**
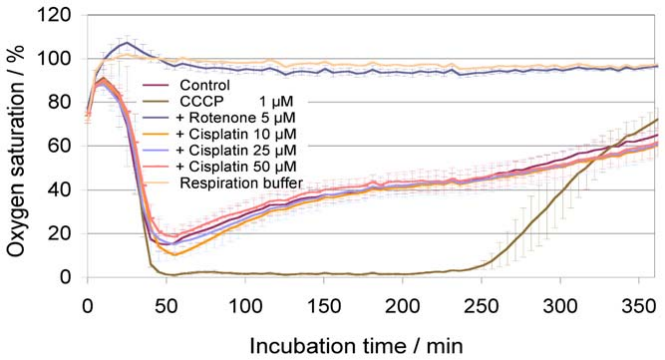
Respiration of isolated mouse liver mitochondria. Mitochondrial respiration consumes oxygen, depicted as a decrease of oxygen saturation over time. After about 50 min mitochondrial respiration buffer is exhausted, leading to continues reduction of mitochondrial respiratory activity. activity leads to a decrease in oxygen saturation, which decreases over time (control). Inhibition of mitochondrial activity blocks oxygen consumption, resulting in continuous high oxygen concentration. Controls: (*i*) **“Control”**: respiration buffer without cisplatin; (*ii*) **“CCCP”**: carbonyl cyanide 3-chlorophenylhydrazone uncouples respiration leading to maximum consumption and complete depletion of oxygen (0%); (*iii*) **“Rotenone”**: an inhibitor of respiratory chain complex I, completely abolishes mitochondrial respiration leaving oxygen concentration unchanged at 100%; (*iv*) **“Respiration buffer”**: respiration buffer without mitochondria and without cisplatin. In **Cisplatin** samples, cisplatin was added at the start of measurement at indicated concentrations (5 µM, 10 µM, 25 µM, 50 µM). No difference between control (without cisplatin) and cisplatin treatment is visible.

Because of the immediate effect of cisplatin on respiration of colon cancer and liver cancer cell lines, we decided to analyze whether cisplatin directly affects mitochondrial respiration using a mitochondrial activity assay with isolated mouse liver mitochondria (Fig. 4). While the well-established mitochondrial inhibitor rotenone and the uncoupler CCCP clearly blocked and increased mitochondrial activity, respectively, cisplatin had no effect on mitochondrial respiration within 4–5 h, independent of the cisplatin concentration used. This clearly shows that respiratory changes observed with intact cells in the cell biosensor system must have resulted from a cellular response to cisplatin treatment.

**Figure 5:**
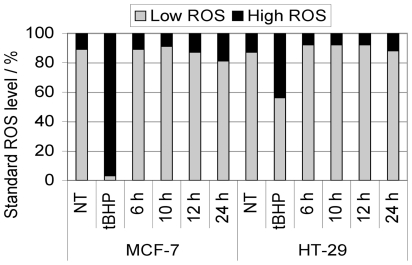
Intracellular ROS formation. MCF-7 and HT-29 were treated with 50 µM cisplatin for the indicated incubation times (6 h, 10 h, 12 h, and 24 h). As positive control, cells were treated with 500 µM *t*BHP (*tert*-butyl hydroperoxide) for 3 h. No significant induction of intracellular ROS with 50 µM cisplatin was observed at the analyzed time points.

We therefore analyzed the formation of ROS in response to cisplatin treatment after 6, 10, 12, 24 h in MCF-7 and HT-29 cells, assuming that ROS could be a mediator of early cisplatin response, including respiration change, and also an important mediator of cisplatin-induced cell death [10]. Surprisingly, no significant ROS formation could be detected in MCF-7 and HT-29 cells treated with 50 µM cisplatin (Fig. 5).

**Figure 6:**
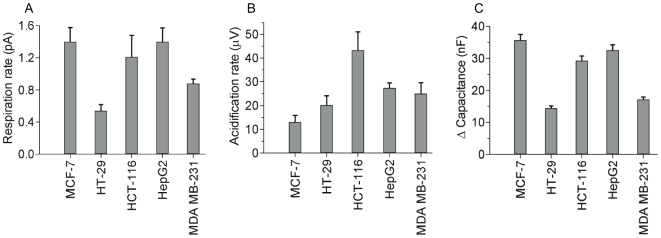
Cell-type specific basal levels of metabolism and cell layer impedance. Basal level of oxygen consumption, acidification of medium, and cell impedance recorded in the cell biosensor chip without drug treatment and at the beginning of experiments before treatment with cisplatin; *A*, basal respiratory activity (change of oxygen of medium); *B*, basal glycolytic rate (change of extracellular pH); *C*, relative difference of electrical capacity in comparison to cell-free biosensor chip.

Glycolysis decreases after 6–7 h in the colorectal cancer cell line HCT-116 and in HepG2 or after 9–10 h in the colorectal cancer cell line HT-29. This is slightly before or at about the same time when impedance decreased. In striking contrast, glycolysis was stimulated in the breast cancer cell line MCF-7 around 8 h after start of treatment, clearly before cells started to die in the biosensor assay. This pronounced difference between MCF-7 and all other cell lines led us to analyze the basic metabolic activity. Raw values of basic respiratory and glycolytic activity show that MCF-7 cells had the lowest glycolytic but the highest respiration rate. In contrast, HT-29 had the lowest respiration and a higher glycolytic activity compared to MCF-7 (Fig. 6).

### Cell signaling and gene expression suggest a transition from general stress response to pro-apoptotic signaling

**Figure 7:**
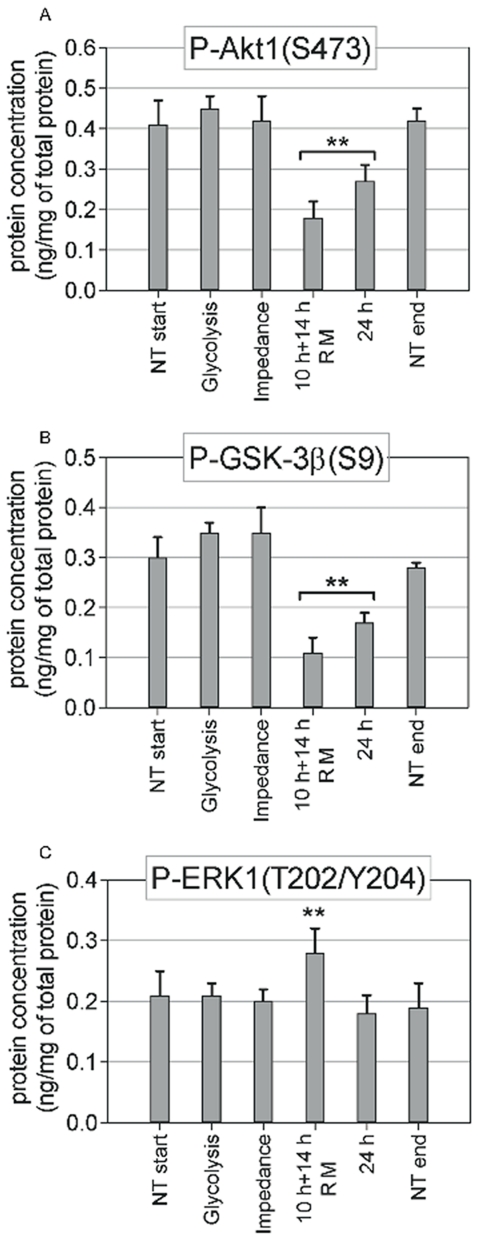
Change in Akt1 (*A*), GSK-3β (*B*), and ERK1 (*C*) pathway activity. Concentrations of selected phosphoproteins were measured with an ELISA microarray in MCF-7 cells at the time points when glycolysis and impedance were significantly changed with 50 µM cisplatin treatment, and after 24 h with a 10-h pulse with 50 µM cisplatin (followed by 14 h with cisplatin-free medium [RM]) or after continued treatment for 24 h with 10 µM cisplatin. For control, the concentration of phosphoproteins was measured in untreated cell samples at the same time points when glycolysis changed in treated samples (NT start) and at the end of experiments (24 h, NT end).

To obtain a more detailed picture of the cellular response of MCF-7 cells to cisplatin, we analyzed the phosphorylation of key signal transduction mediators (Fig. 7*A–C*) and gene expression profiles directly from the samples analyzed in the biosensor chip immediately after the first change in (*i*) glycolytic rate and (*ii*) impedance became visible when treated with 50 µM cisplatin. Our results do not show any significant change in activation of Akt1, GSK-3β, and ERK1/2 MAP-kinases within the first 8–11 h (glycolysis and impedance change) of cisplatin treatment. After 24 h a significant decrease in p-Akt1 and p-GSK-3β reflects reduced pro-survival signaling, which was even more pronounced after a shorter pulse with 50 µM cisplatin than with continuous lower (10 µM) treatment (Fig. 7). The specific efficiency of 50 µM cisplatin treatment is also supported by an increased level of p-ERK1, which was shown to contribute to apoptosis induction by cisplatin [11].

**Figure 8:**
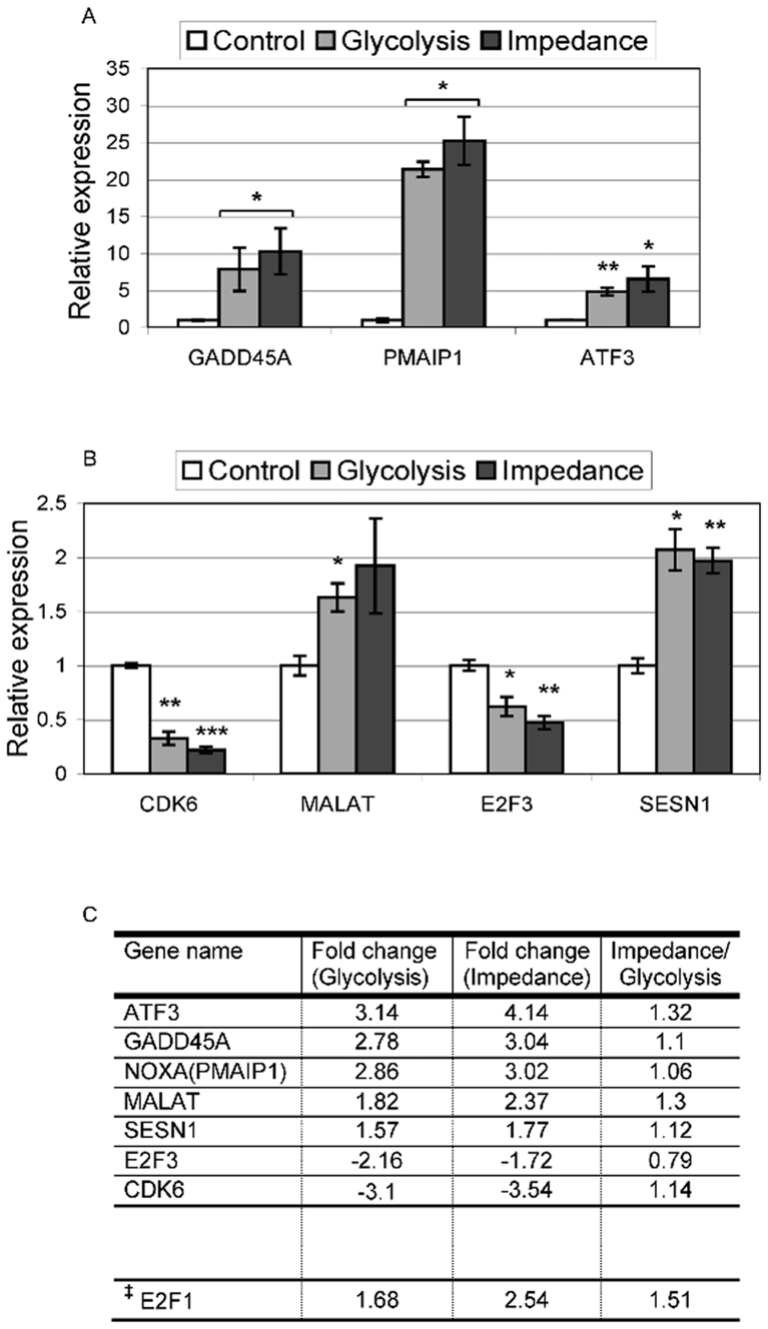
Changes in gene expression of key regulators at the time of glycolysis and impedance change in MCF7 cells. To confirm large-scale gene expression profiling data, selected genes with a focus on p53 signalling were analyzed by quantitative real-time RT-PCR using independent samples. *A–B*, real-time RT-PCR shows strong induction of GADD45A, PMAIP1 and ATF3 (*A*) and low, but significant regulation of MALAT and SESN1 (up) and CDK6 and E2F3 (down) (*B*) Control: samples from incubation on biosensor chip without cisplatin treatment; Glycolysis: samples from biosensor chip at time of glycolysis change (Fig. 1*D*); Impedance: samples from biosensor chip at time of impedance change (Fig. 1*E*). Footnote to Fig. 8. Significance values: * p<0.05; ** p<0.01; *** p<0.001. *C*, results from gene expression profiling for the same genes. ^‡^E2F1 is included because it shows similar significance in micro array data as E2F3. Fold change relative to control sample for glycolysis and impedance change. Impedance/Glycolysis: relative ratio of changes in glycolysis and impedance.

This specific pro-apoptotic regulation is also confirmed by changes in gene expression recorded at the time of glycolysis and impedance change (GEO NCBI Accession number: GSE28274). Using a 1.75-fold change and a minimal normalized signal of 100 as cutoff we obtained 1338 significantly regulated probe sets, of which 335 were up- and 1003 down-regulated (supplemental Table S1). Most genes were differentially expressed at both time points, but a significant number was stronger regulated at the later time point of impedance change. Top-regulated genes for each treatment are shown in supplemental Table S2. While strongly regulated genes at glycolysis change, were not further induced, the top list from impedance change shows several genes significantly more changed within the additional (about) 2 h between the two time points analyzed before cells started to die (impedance change). The reliability of the gene expression profiles was confirmed by quantitative real-time RT-PCR analysis of selected genes in independent experiments (Fig. 8*A–B*).

Further analysis by gene ontology and pathway annotation using DAVID [12], [13] showed an enrichment of genes associated with a pro-apoptotic transition. Genes up-regulated at change of impedance were clearly enriched for p53-signaling (p = 0.0023), cell cycle control (cyclin-dependent protein kinase activity: p = 0.001), and programmed cell death (apoptosis: p = 0.0058). Down-regulated genes were enriched for various cell proliferation-associated cell processes, in particular, wnt (p = 0.000008), tgfβ (p = 0.0019), MAP-kinase (p = 0.0016), hedgehog (p = 0.0033), and erbb (p = 0.000082) signaling (supplemental Table S3).

The pro-apoptotic regulation is well reflected in the main regulatory circuit of p53 control, with up-regulation of E2F1, but down-regulation of E2F3 (Fig. 8*C*). Analysis of genes already significantly induced at glycolysis, suggests that at first a general stress response including cell cycle arrest is activated, before induction of cell death occurs; gene groups significantly up-regulated at glycolysis change include: response to protein stimulus, regulation of cyclin-dependent protein kinase activity, stress response, response to organic substance (p<0.05). The complex regulatory activities, from initial stress response and transition to apoptosis, are also reflected in the strong induction of p53 signaling mediators (Fig. 8), namely ATF3 a mediator of p53 signaling, the pro-apoptotic proteins TSP1, maspin, NOXA(PMAIP1), and the cell cycle regulator GADD45A, as well as the down-regulation of CDK6, required for cell cycle progression.

## Discussion

In this study we used a novel cell biosensor chip to follow, in real time, the metabolic response to cisplatin, one of the best-studied anticancer agents. Cisplatin treatment leads to immediate as well as delayed metabolic adaptations (measured as respiration and acidification) and changes in cell–cell interaction and adhesion (measured as impedance). The first cellular response observed after initiation of treatment provides a specific time-resolved insight in cisplatin toxicity and hints to the possible molecular mechanism.

We performed real-time measurements of five cancer cell lines: two types of breast cancer cell lines (MCF-7 and MDA-MB-231), two types of colorectal cancer cell lines (HT-29 and HCT-116), and the liver hepatocellular carcinoma HepG2. In all cells, oxygen consumption was the first parameter to be affected by cisplatin, either almost immediately after cisplatin treatment started, as in HT-29, HCT-116, HepG2, and MDA-MB-231, or after 5–6 h in the case of MCF-7 cells. This appears to confirm the hypothesis that mitochondria are a primary target of cisplatin-mediated DNA damage [14], [15], indicating that cisplatin accumulates in mitochondria, binds to mitochondrial DNA (mtDNA), and causes mitochondrial damage.

Our results with isolated mitochondria showed no immediate effect on mitochondrial activity in a time frame when respiration of intact cells is clearly reduced. This correlates with previous observations that lower concentrations of cisplatin do not inhibit activity of isolated rat liver mitochondria [14]. Thus, the immediate respiratory response to cisplatin must result from a mitochondrial activity-independent mechanism involving cellular signaling or DNA damage. However, because it is also observed in the p53 mutant cell line MDA-MB-231, DNA damage-mediated signaling may not be the primary cause.

Glycolysis, on the other hand, decreases after 6–7 h in the colorectal cancer cell line HCT-116 and in HepG2 cells which occurs at about the same time or slightly before impedance decreased. In striking contrast, glycolysis was stimulated in the breast cancer cell line MCF-7 around 8 h after start of treatment, clearly before cells started to die in the biosensor assay. No change in glycolysis was observed in the cisplatin-resistant cell line MDA-MB-231. The pronounced difference between MCF-7 and all other cell lines led us to analyze the basic metabolic activity.

Raw values of basic respiratory and glycolytic activity show that MCF-7 has the lowest acidification and highest respiration rate. In contrast, HT-29 had the lowest respiration but higher glycolytic activity compared to MCF-7. Other stress-inducing compounds like methyl methanesulfonate (MMS) and *tert*-butyl hydroperoxide (*t*BHP) also showed that MCF-7 cells are the only cells among the cell lines tested in this work that were able to enhance glycolysis when respiration was inhibited, while HT-29 cells were able to increase respiration in stress conditions (supplemental Fig. S1 *A*–*L*). We also showed previously that MCF-7 and HT-29 cells respond differently to other cytotoxic compounds regarding glycolysis and respiration [16], [17].

Impedance measurements reflect cell morphological and cell adhesion changes, e.g., cell-cell and cell-matrix contacts. MCF-7 cells treated with cisplatin showed a rapid decline of impedance after 10–11 h in a dose-dependent manner. In contrast to MCF-7 cells, HCT-116, HepG2, and HT-29 display a lower rate of impedance decrease (lower slope) which could indicate lower sensitivity to cisplatin. This may be explained by a higher basic levels of glycolysis. In a recent study centered on cisplatin and 2-deoxy-D-glucose cotreatment, it was suggested that a high basal glycolytic rate correlates with a more cisplatin-resistant phenotype [18]; our results seem to confirm this observation. Although the rate of impedance reduction is different, all cell lines showed ongoing cell death when cisplatin was removed after 24 h, indicating persistent cellular damage. This is in good agreement with DNA damage being the main mode of cisplatin action [3], [4], [5], [6].

Even after exposure for only 6 h, MCF-7 cells start to die in the same time frame and do not escape cisplatin-induced cell death (data not shown).

Cisplatin treatment also had been associated with specific activation of signaling pathways mediating DNA damage response and cellular proliferation, including p53, MAP-kinases ERK1/2, and Akt1 [19], [20], [21]. It has been shown that ERK1 contributes to apoptosis by activating the tumor suppressor p53 [11], [22]. In our experiments no significant change in these key signaling pathways was observed within the first 8–11 h of cisplatin treatment. Only after 24 h p-Akt1 and p-GSK-3β levels were reduced, and p-ERK1 increased at the higher cisplatin concentration. Thus, these signaling pathways are not primary mediators of cisplatin-induced cell death but rather mirror ongoing cell death.

The specific pro-apoptotic regulation is also confirmed by changes in gene expression, which include a clear induction of pro-apoptotic genes involved in p53-mediated cell fate decision, such as TSP1, maspin, PMAIP1(NOXA), and GADD45a. Expression of these genes was already induced when glycolysis changed and for some genes increased even further until onset of cell death. In contrast, stress response genes including HSP70, DUSP1, ID2, and CYR61 were strongly regulated at glycolysis change without further induction. This suggests that in response to cisplatin, first an initial general stress response is activated before pro-apoptotic cell fate decision occurs. The time frame observed in our online measurements indicates that this crucial transition occurs around 10 h after onset of cisplatin treatment. Surprisingly, this is in good agreement with a recently published model of p53-dependent cell fate decision in response to DNA damage [23]. Our results also show that p53 target genes are involved in this cell fate decision. Furthermore, the primary p53 regulatory circuit is clearly modulated towards p53 activation and pro-apoptotic signaling by E2F1 and E2F3, the one by an up-regulated and the other by a down-regulated mechanism.

In essence, for the first time we have been able to show a clear time line for cisplatin-induced cell death in different cancer cell lines and to confirm once more that the p53 regulatory circuit is crucial for this cell fate decision, which is further supported by the good agreement of our time line measurements with modeling of the p53 damage response.

## Materials and Methods

### Real-time monitoring of cellular metabolism in living cells

Changes in cellular metabolism and morphology were analyzed using a Bionas 2500 sensor chip system (Bionas, Rostock, Germany). The sensor chips (SC1000) in the Bionas 2500 allow the continuous measurement of three important parameters of cellular metabolism: (*i*) oxygen consumption using Clark-type electrodes, (*ii*) change in the pH of the extracellular environment using ion-sensitive field effect transistors, (*iii*) and the impedance between two interdigitated electrode structures to register the impedance under and across the cell layer on the chip surface. Before measurement, cells were seeded on the sensor chip in DMEM with penicillin/streptomycin and 10% (v/v) FCS (PAA) and incubated in a standard tissue culture incubator at 37°C, 5% CO_2_, and 95% humidity for 24 h until 80–90% confluence was reached. Sensor chips with cells were then transferred to the Bionas 2500 analyzer in which medium is continuously exchanged in 8-min cycles (4 min exchange of medium and 4 min without flow) during which the parameters were measured. The running medium (RM) used during analysis was DMEM without carbonate buffer (PAN Cat. Nr. P03-0010) and only weakly buffered with 1 mM Hepes and reduced FCS (0.1%) and low glucose (1 g/L). For drug activity testing, the four following steps were included: (a) 5 h of equilibration with only running medium with 4 min stop/flow incubation intervals, (b) drug incubation with compounds freshly dissolved in medium at indicated concentrations also with 4 min stop/flow, and (c) a drug free step in which cells are again fed with RM without compound; (d) at the end of each experiment, cell layers are removed by adding 0.2% Triton X-100 to obtain basic signals without living cells on the sensor surface as negative control. Before each experiment, the tubes of the system were cleaned, washed with 70% EtOH and then rinsed with PBS and RM.

### Cell culture

The cell lines that were used included HT-29, HCT116, HepG2, MDA-MB-231, and MCF-7 (all from ATCC); all were cultured in Dulbecco's modified eagle medium (DMEM) (PAA, Pasching, Austria), supplemented with 10% FBS (PAA, Pasching, Austria), 1% penicillin/streptomycin (Gibco Invitrogen) within a CO_2_ incubator at 37°C with 5% CO_2_. For the MDA-MB-231 cell line we have used Leibovitz's L-15 Medium (Gibco Invitrogen) and non-essential amino acids (Gibco invitrogen). All cell lines were seeded at a density of 2×10^5^ in each chip in 450 µL DMEM except MCF-7 with 1.5×10^5^ per chip. After seeding cells were grown on the chip for 20–24 h in standard cell culture conditions to approximately 80–90% confluence on the chip surface at the time of measurement.

### Measurement of mitochondrial oxygen consumption

The measurement was performed using OxoPlate® (PreSens, Germany), 96-well plates which contain an immobilized oxygen sensor at the bottom of each well. Fluorescence is measured in dual mode, excitation 540 nm and emission 650 nm, with reference emission 590 nm. Their signal ratio 650/590 nm corresponds to the oxygen partial pressure. The calibration of the fluorescence reader is performed using a two-point calibration with oxygen-free water (1% Na_2_SO_3_) and air-saturated water with oxygen partial pressure corresponding to 0% and 100%, respectively. 18 µg of freshly isolated mitochondria were suspended in 100 µL of Respiration Buffer (25 mM sucrose, 100 mM KCl, 75 mM mannitol, 5 mM MgCl_2_, 10 mM KH_2_PO_4_, 0.5 mM EDTA, 10 mM Tris, 0.1% fatty acid-free BSA, pH 7.4) containing 10 mM pyruvate, 2 mM malate, 2 mM ADP, and 0.5 mM ATP to activate oxidative phosphorylation. Cisplatin was diluted so that the appropriate concentration in the wells was established after adding the mitochondria. Fluorescence was measured continuously for 400 min with kinetic intervals of 5 min by a Tecan Safire^2^ (Tecan, Maennedorf, Switzerland) microplate reader at 37°C. During the measurements the plates were sealed with a breathable membrane (Diversified Biotech, Boston, MA). Additional controls were 5 µM rotenone (Sigma-Aldrich) as inhibitor of respiratory chain complex I and 1 µM CCCP (carbonyl cyanide 3-chlorophenylhydrazone, Sigma-Aldrich) as uncoupling agent, capable of increasing electron flow through the respiratory chain thereby increasing the oxygen consumption.

### Antibodies and recombinant protein standards for protein microarray

Antibody microarrays [24] based on ArrayTube™ platform (Clondiag, Jena, Germany) utilize isotype-specific capture antibodies and biotinylated phospho-specific detection antibodies against 5 human/mouse/rat proteins of interest: phospho-GSK-3β(S9), phospho-Akt1(S473), phospho-ERK1(T202/Y204), phospho-ERK2(T185/Y187), and phospho-CREB(S133). The microarrays used also antibody pair against human paxillin. Microarray calibration was performed with recombinant protein standards. The antibodies and calibration standards were obtained from R&D Systems, Minneapolis, MN, USA (DuoSet® IC kits). MCF-7 cell protein lysates were isolated directly from the sensor chip at time points when changes in glycolysis and impedance occur, or at other time points indicated. Protein quantification was done as described [25].

### RNA isolation, Affymetrix gene-chip hybridization, reverse transcription and real-time PCR

Total RNA was isolated directly from MCF-7 cells treated in the Bionas system at time points when changes in glycolysis and impedance occur using RNeasy Mini Kit (Qiagen) according to the manufacturer's instructions. RNA quality was examined by agarose gel electrophoresis and concentration was determined by UV absorbance. Affymetrix array analysis (GeneChip® U133 2.0plus Human Genome) was performed according to the manufacturer's protocol. Gene expression profiles were analyzed using the dChip software [26] and rank based normalization [27]; assignment of functional groups was done using DAVID Bioinformatics Resources 6.7 (Database for Annotation, Visualization, and Integrated Discovery) of NIAID (NIH) [12], [13] and free word data mining. For quantitative RT-PCR 250 ng of total RNA was reverse transcribed using oligo(dT)_18_ primer (0.5 mg/mL, Fermentas) and random hexamers primer (100 µM, Fermentas) and RevertAid™ Premium Enzyme Mix. Gene expression was assayed by quantitative real-time PCR on LightCycler® 480 (Roche Applied Science) using LightCycler® 480 SYBR Green I Master with 1 µL cDNA (1∶5 dilution of transcribed cDNA) and the primers listed in Table 2.

**Table 2:** List of forward and reverse primers used to perform quantitative real-time PCR.

Primer name	Primer sequence
ATF3-5s	5′ – CATCCAGAACAAGCACCTC – 3′
ATF3-3as	5′ – GCATTCACACTTTCCAGC – 3′
CDK6-5s	5′ – GATGTGTGCACAGTGTCACGAAC – 3′
CDK6-3as	5′ – GTGGTTTTAGATCGCGATGCAC – 3′
E2F3-5s	5′ – GTTCATTCAGCTCCTGAGCCAG – 3′
E2F3-3as	5′ – CACTTCTTTTGACAGGCCTTGAC – 3′
SESN1-5s	5′ – CTGAAGAGCATCCAGGAAC – 3′
SESN1-3as	5′ – GCAGTAGATAGTGCTGAG – 3′
MALAT-5s	5′ – GGATCCTAGACCAGCATGC – 3′
MALAT-3as	5′ – GGTTACCATAAGTAAGTTCCAG – 3′
GADD45A-5s	5′ – CGATAACGTGGTGTTGTGC – 3′
GADD45A-3as	5′ – GAATGTGGATTCGTCACC – 3′
PMAIP1-5s	5′ – ATGCCTGGGAAGAAGG– 3′
PMAIP1-3as	5′ – CAGGTTCCTGAGCAGAAG– 3′
Humbetaactin-5s	5′ – CTGACTACCTCATGAAGATCCTC– 3′
Humbetaactin-3as	5′ – CTGCTGAAGAAGCACATCGATTC– 3′

### Intracellular ROS determination

MCF-7 and HT-29 cell lines (10^5^ cells/mL medium) were treated with 50 µM cisplatin for 6, 10, 12, and 24 h. After treatment they were trypsinized and resuspended in 1 mL of Dulbecco's PBS (Gibco) supplemented with 1% bovine serum albumin (PAA Laboratories). 10^5^ cells were then incubated for 15 min in the dark with 10 µM dihydroethidium. Accumulation of reactive oxygen species (ROS) was measured by flow cytometry using a FACSCalibur instrument (Becton Dickinson). Excitation and emission settings were 488 nm and 564–606 nm (FL2 filter), respectively. Results are presented as a percentage of cells that showed physiological levels of ROS (low ROS) and a percentage of cells with a high level of ROS.

## Supporting Information

Figure S1 **Real-time response profiles of cellular respiration, glycolysis, and impedance upon MMS or tBHP treatment.** **MCF-7** breast cancer cells or **HT-29** colon carcinoma cells were treated with the strong stress inducing agents methyl methanesulfonate (MMS) and *tert*-butyl hydroperoxide (tBHP). MMS is a methylating agent which leads to strong intracellular ROS formation upon methylation of DNA; tBHP is an organic peroxide that leads to peroxide formation in the medium. Treatment started after 5 h of equilibration of cancer cell cultures in the biosensor chip system and continued over 6 h. **RM** (running medium) marks time with medium without compound; exposure period when compounds were present at indicated concentrations. (**A,D,G,J**) change in respiration (oxygen in the medium), (**B,E,H,K**) glycolysis, depicted as change in acidification of the medium (extracellular pH), (**C,F,I,L**) change in impedance of cell layer (potential between interdigitated electrodes). MCF-7 treated with MMS (A–C); MCF-7 treated with tBHP (G–I); HT-29 treated with MMS (D–F); HT-29 treated with tBHP (J–L).
(TIF)

Table S1 Genes regulated by cisplatin in MCF-7 cells at change of (i) glycolysis (8–9 h) and (ii) impedance (10–11 h).
(XLS)

Table S2 Genes regulated by cisplatin in MCF-7 cells at change of (i) glycolysis (8–9 h) and (ii) impedance (10–11 h).
(XLS)

Table S3 Significantly regulated functional groups*.
(XLS)
