# Supplementary figures and images for: Real-Time Monitoring of Cisplatin-Induced Cell Death
Source: PLoS One. 2011 May 16;6(5):e19714. doi: 10.1371/journal.pone.0019714 (PMC3095603; doi:10.1371/journal.pone.0019714)

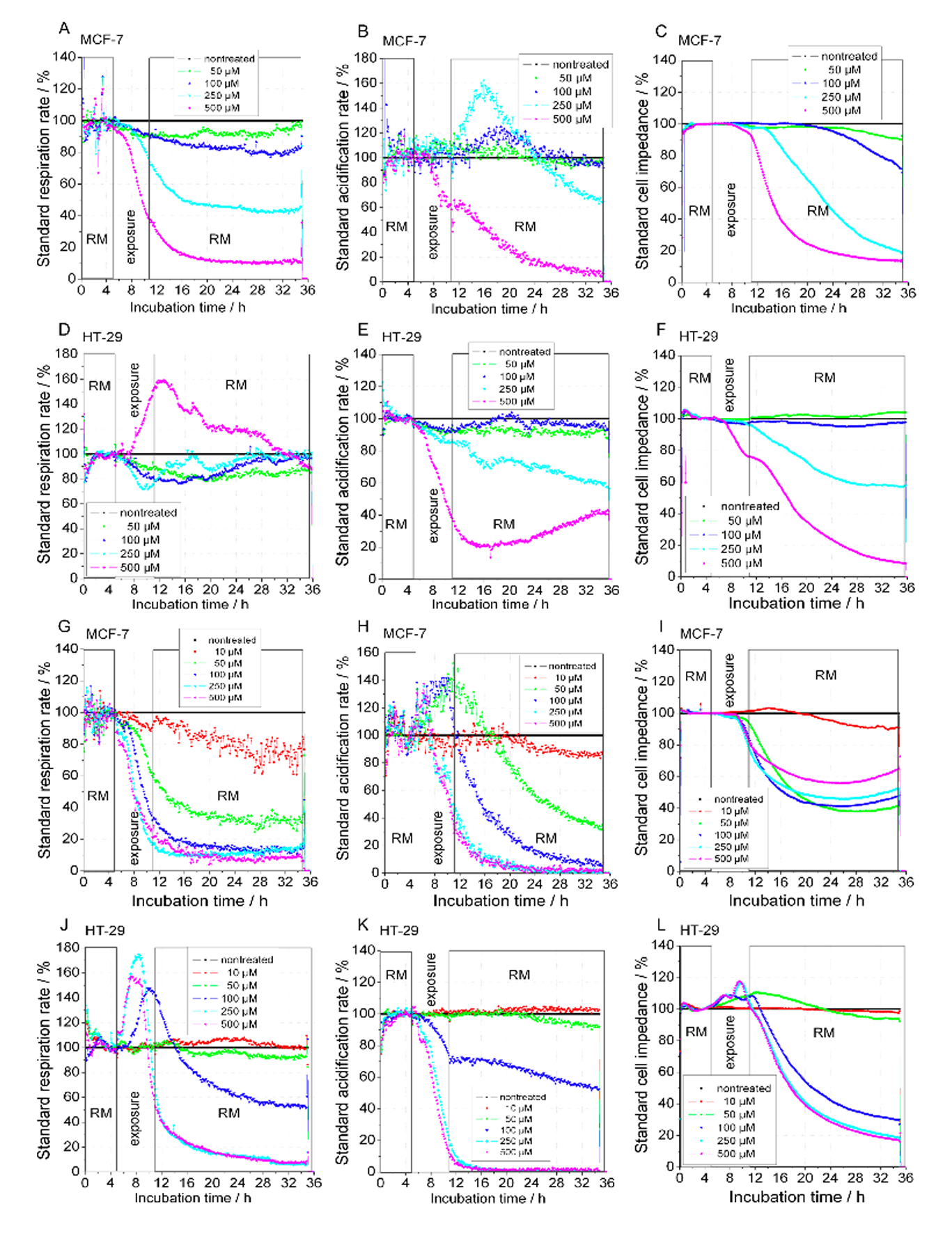

Supplement: Figure S1 — Real-time response profiles of cellular respiration, glycolysis, and impedance upon MMS or tBHP treatment. MCF-7 breast cancer cells or HT-29 colon carcinoma cells were treated with the strong stress inducing agents methyl methanesulfonate (MMS) and tert-butyl hydroperoxide (tBHP). MMS is a methylating agent which leads to strong intracellular ROS formation upon methylation of DNA; tBHP is an organic peroxide that leads to peroxide formation in the medium. Treatment started after 5 h of equilibration of cancer cell cultures in the biosensor chip system and continued over 6 h. RM (running medium) marks time with medium without compound; exposure period when compounds were present at indicated concentrations. (A,D,G,J) change in respiration (oxygen in the medium), (B,E,H,K) glycolysis, depicted as change in acidification of the medium (extracellular pH), (C,F,I,L) change in impedance of cell layer (potential between interdigitated electrodes). MCF-7 treated with MMS (A–C); MCF-7 treated with tBHP (G–I); HT-29 treated with MMS (D–F); HT-29 treated with tBHP (J–L). (TIF) [file pone.0019714.s001.tif]
